# Supplementary material for: Increased levels of immature and activated low density granulocytes and altered degradation of neutrophil extracellular traps in granulomatosis with polyangiitis
Source: PLoS One. 2023 Mar 15;18(3):e0282919. doi: 10.1371/journal.pone.0282919 (PMC10016653; doi:10.1371/journal.pone.0282919)
Supplement: S1 Fig — A) Quantification of NDGs. (A) Frequencies of NDGs among PBMCs and (B) absolute numbers of NDGs were determined by flow cytometry (HD, n = 21; GPA, n = 12; SLE, n = 21) using the Truecount method. NDGs were identified as CD16+SSC+ cells in whole blood. Each dot represents one measured patient sample. Patients receiving prednisolone dosages of ≥ 20mg daily (3 patients with GPA and one patient with SLE) are indicated by triangles. Median values ± IQR are presented. Data were analyzed by Kruskal-Wallis-test, no significant differences were determined. B) Microscopy images of netting neutrophils. Representative immunofluorescence images to identify NETs formed by normal-density granulocytes (NDG) from one healthy donor and one patient with GPA without stimulation (upper row) or after certain time-points after PMA stimulation. (PDF) [file pone.0282919.s001.pdf]

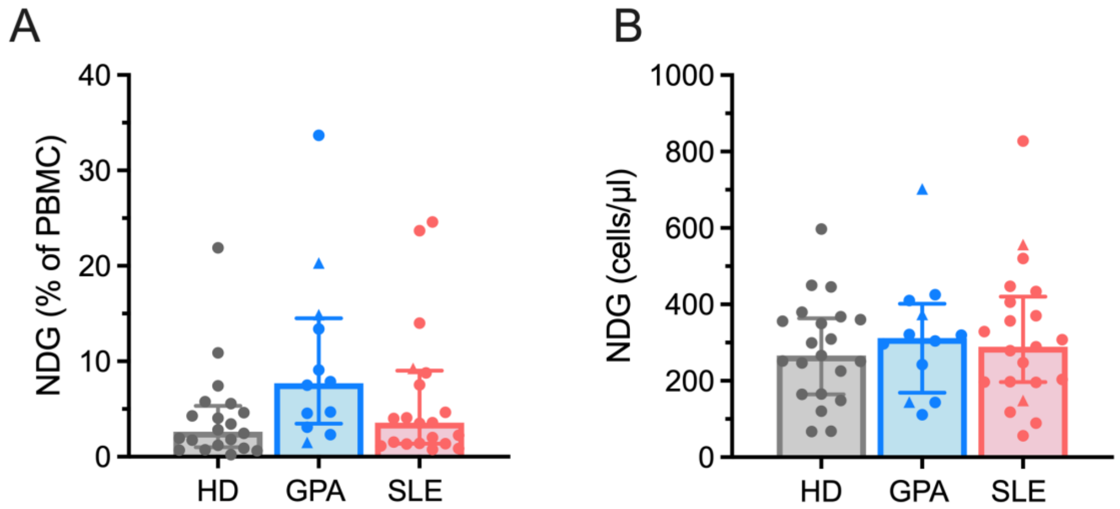

Figure S1A: Quantification of NDGs: *(A) Frequencies of NDGs among PBMCs and (B) absolute numbers of NDGs were determined by flow cytometry (HD, n=21; GPA, n=12; SLE:n=21) using the Truecount method. NDGs were identified as CD16⁺SSC⁺ cells in whole blood. Each dot represents one measured patient sample. Pateints receiving prednisolone dosages of ≥ 20mg daily (3 patients with GPA and one patient with SLE) are indicated by triangles. Median values ± IQR are presented. Data were analysed by Kruskal-Wallis-test, no significant differences were determined.*

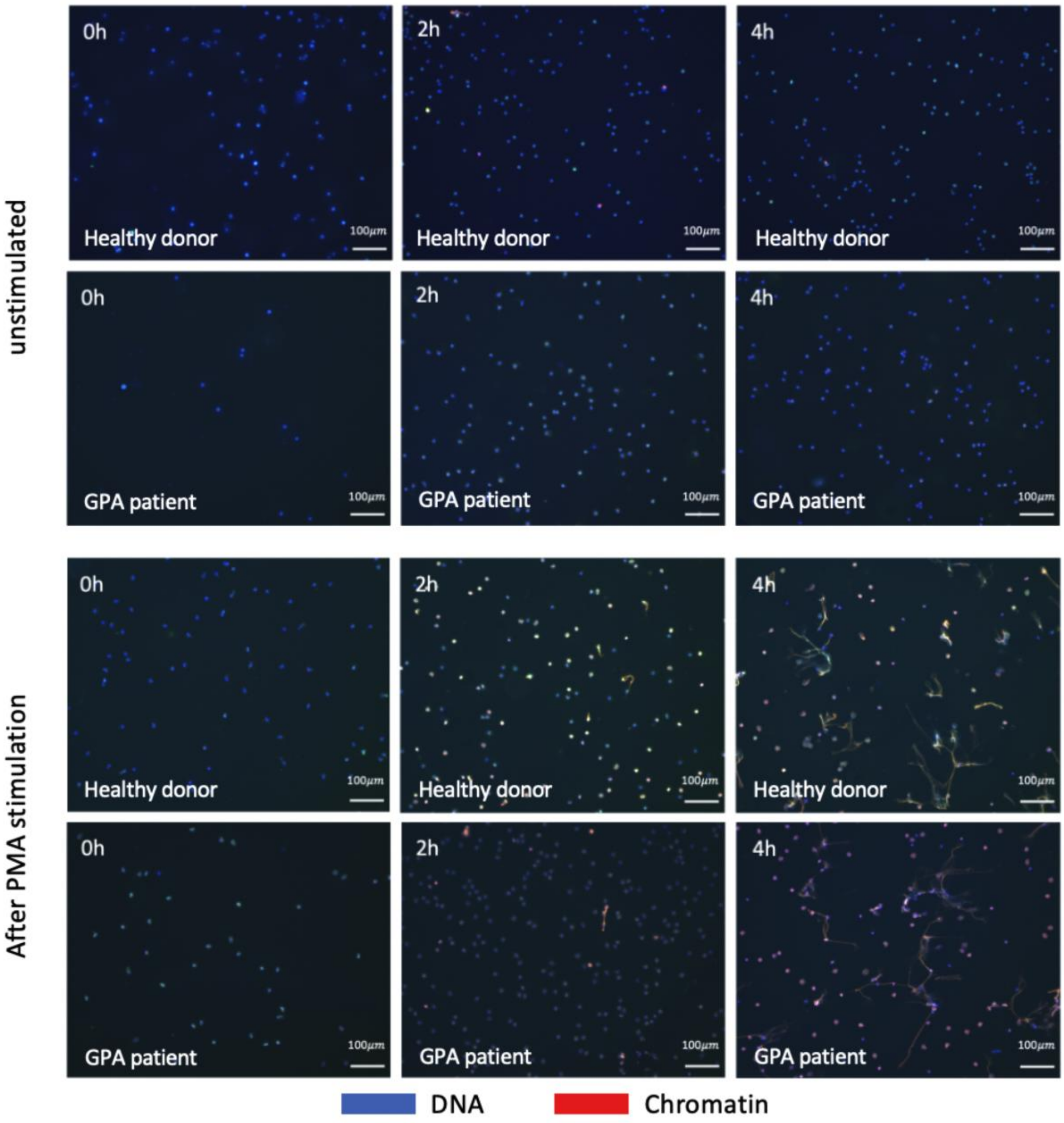

Figure S1B: Microscopy images of netting neutrophils. *Representative immunoflourescence images to identify NETs formed by normal-density granulocytes (NDG) from one healthy donor and one patient with GPA without stimulation (upper row) or after certain time-points after PMA stimulation.*
