# Supplementary material for: Increased levels of immature and activated low density granulocytes and altered degradation of neutrophil extracellular traps in granulomatosis with polyangiitis
Source: PLoS One. 2023 Mar 15;18(3):e0282919. doi: 10.1371/journal.pone.0282919 (PMC10016653; doi:10.1371/journal.pone.0282919)
Supplement: S1 Table — (PDF) [file pone.0282919.s002.pdf]

Table S2: Patient characteristics in which NETs have been investigated.

	Healthy donors	GPA	SLE	*P* Value
**Number**	9	10	14	
**Age (median, IQR)**	26 (22-29)	66 (57-70)	36 (30-46)	HD vs. GPA *P*<0.001<br>HD vs. SLE *P*=0.002
**Female (n, %):**	8 (88.89)	4 (40.0)	13 (92.86)	HD vs. GPA *P*<0.057<br>HD vs. SLE *P*=1.00
**Clinical manifestations (n, %)**				
**Renal involvement**	0	8 (66.7)	3 (21.4)	
**Head/Neck**	0	7 (58.3)	0 (0)	
**Pulmonary**	0	8 (66.7)	0 (0)	
**ANCA status**				
**c-ANCA positivity (n, %)**	-	9 (90.0)	0	
**p-ANCA positivity (n, %)**	-	0 (0)	0	
**BVAS (median score, range)**	-	6.5 (0-18)	-	
**SLEDAI-2K (median score, range)**	-	-	4 (2-24)	
**Immunosuppressive and biologic medications (n, %)**	0	GC: 10 (100)<br>AZA: 2 (20)<br>MTX: 5 (50)<br>RTX: 3 (30)	GC: 12 (85.7)<br>HCQ: 9 (64.3)<br>AZA: 5 (35.7)<br>MMF: 2 (14.3)<br>MTX: 2 (14.3)<br>CsA: 0 (0.0)<br>BEL: 0 (0.0)	
**Daily prednisolone dosage (mg) (median, range)**	0	4.5 (2-50)	8 (0-20)	

Demographic and serological data of GPA and SLE patients, in which NETs have been investigated. Abbreviations: AZA, Azathioprine; BEL, Belimumab; BVAS, Birmingham Vasculitis Activity Score; CsA, Ciclosporin A; GC, Glucocorticoids; HCQ, Hydroxychloroquine; MMF, Mycophenolate Mofetil; MTX, Methotrexate; RTX, Rituximab; SLEDAI-2K, Systemic Lupus Erythematosus Disease Activity Index 2000. Statistical analysis comparing differences in age was performed using the Mann-Whitney test, sex differences with the chi-square test.
