# Supplementary material for: Increased levels of immature and activated low density granulocytes and altered degradation of neutrophil extracellular traps in granulomatosis with polyangiitis
Source: PLoS One. 2023 Mar 15;18(3):e0282919. doi: 10.1371/journal.pone.0282919 (PMC10016653; doi:10.1371/journal.pone.0282919)
Supplement: Attachment — Submitted filename: Response to Reviewers_R1.docx [file pone.0282919.s003.docx]

*Rebuttal Letter*

January 31st, 2023

**PONE-D-22-27387**

**Increased levels of immature and activated low density granulocytes and altered degradation of neutrophil extracellular traps in granulomatosis with polyangiitis**

**PLOS ONE**

Dear Reviewer,

We are grateful for your critical remarks and suggestions. We revised our manuscript accordingly. Please find below our response to each point you raised.

1. The cohorts should be separated into those with active and remission disease (the BVAS range included 0 so remission patients were apparently included). It is likely that disease activity (and, more importantly, the high dose corticosteroids associated with this active disease, will have a big effect on NETosis).

Response: We fully agree with your notion. In fact, we had similar discussions in our group on how to evaluate the effect of disease activity on our readouts. Finally, instead of separating the patients into two separate cohorts, i.e. those with inactive vs. active disease, we decided to capture this issue rather in correlation analysis, as there is no clear clinically significant cut-off, except for BVAS 0 (remission), which was present only in 1 case. As you can see in Fig 5, BVAS scores showed indeed some correlations in major domains, most notably compared to % of NET degradation. For completion, we now added a correlation between BVAS, PR-3 levels with the capacity of NGDs to form NETs (Fig. 5C). Further down the line, we also included the correlation of these results with daily prednisolone dosage as suggested.

2. Note that, although table 1 describes the full cohort, studies examining NETs had fewer participants; these cohorts should be described in a supplementary table.

Response: According to your suggestions we now added a separate table (Tab. S2) that describes the patient characteristics in the cohort of patients from which NETs have been investigated.

3. Fig S1A: it’s not clear what the y-axis represents. Neutrophils in the PBMC layer are LDGs, but the figure refers to NDGs.

The quantification of NDGs was performed by Truecount assessment in full blood samples. We now added a sentence in the method part to clarify.

4. I suggest that, for all figures, the individuals receiving high dose corticosteroids (say >20mg/day) are identified using, for example, open circles. Also, in addition to correlating NET degrading capacity with BVAS, figure 5 should also correlate against steroid dose in both SLE and GPA patients.

Response: We only had three GPA patients and one SLE patient with prednisolone dosages of 20mg daily or above. According to your suggestions, we highlighted those in the Fig 1 and 2 with triangles. In addition, we added in Figure 5 the correlation between steroid usage and outcome parameters (see your comment 1).

5. Are flow cytometry data available for whole blood samples? This is important to clarify the effect of cell isolation on markers such as CD16 and CD63. Indeed, it is not clear why the results pertaining to LDGs are emphasised, given that NETosis, the focus of the paper, was not studied in this cell population.

Response: We thank you for addressing this important issue. In fact, isolation of LDGs from whole blood may alter their phenotype and functional properties. This is exactly the reason why we avoided investigating the capacity of LDGs to form NETs. Another reason is the lack of reliable marker (combinations) to define LDGs in this context and the fact, that many groups already showed enhanced NETosis of LDGs in GPA. We therefore decided to restrict the analysis of LDGs to their surface marker expressions. NDGs had been isolated in a similar way, which justifies comparison of those subsets taking into account our methodological limitations, as depicted in Fig 2.

6. The anti-PR3 antibody levels, rather than c-ANCA levels, should be reported. I presume that none were anti-MPO positive.

Response: Thanks for your important comment. In fact, we measured anti-PR3 antibody levels with ELISA and not just c-ANCA titer. The testkit we use in clinical routine is from ORGENTEC Diagnostika. We now added the detection method in the manuscript and renamed c-ANCA into anti-PR3 in the axis labelling of the figures and, where appropriate, in the manuscript. As assumed, none of the patients was positive for MPO antibodies.

7. Fig 4: There does appear to be a decline in NET degrading capacity with increasing disease activity, although BVAS is a poor means of quantifying the degree of vasculitic injury. Is the same observed with SLEDAI in the lupus participants? Again, it is important to consider the confounding effect of concurrent corticosteroid therapy, which may in fact be the main reason for this decline in NET degradation capacity.

Response: According to your suggestion, we re-analyzed the NET degrading capacity in SLE patients in correlation with clinical (SLEDAI-2K) and serologic (dsDNA-abs) activity markers. As you can see from the results below (Fig.1), there was no clear correlation whatsoever. Therefore, we felt this data would not substantially add valuable information to the manuscript worth showing, if you agree.

Figure 1: Correlation of NET degrading capacity of SLE sera in correlation to clinical (SLEDAI-2K score) and serologic (anti-dsDNA antibody titers) activity markers.

8. The authors comment on some unexpected null findings in the discussion, for example, no increased circulating NETs in GPA. The most likely reason for this is the fact that the study is underpowered, which should be alluded to in the discussion.

Response: Thanks for your suggestion. Indeed, the low number of patients is probably the major limitation of this study, although we already found some significant findings. We modified the discussion accordingly.

9. The methods section contains descriptions of quantification of DCs, B cells and other cell types that have no counterpart in the results section. Were these experiments performed? If not, it is not necessary to describe the respective methods.

Response: We thank for your notion. In fact, phenotypic data on DC and B cell subsets have been included in a previous version of the manuscript but later excluded because they did not add significant findings. Therefore, we deleted this part from the method section.

10. The strongest result is the reduction in NET degradation capacity of GPA serum. Were DNAse-1 levels measured in serum? This would be an obvious next step in determining the mechanism of this observation.

Response: We completely agree with your suggestion. We also thought to determine downstream mechanisms, but unfortunately, the amount of available cryopreserved serum left after previous investigations was not sufficient to analyze DNAse levels in most patients. Nevertheless, reduced DNase 1 activity, as previously reported in AAV (J Am Soc Nephrol 2014).

Sincerely,

Tobias Alexander, MD

Charité – University Medicine Berlin, Department of Rheumatology and Clinical Immunology, Charitéplatz 1, 10117 Berlin, Germany
